# Functional Relevance of Citrulline in the Vegetative Tissues of Watermelon During Abiotic Stresses

**DOI:** 10.3389/fpls.2020.00512

**Published:** 2020-05-05

**Authors:** Qiushuo Song, Madhumita Joshi, James DiPiazza, Vijay Joshi

**Affiliations:** ^1^Department of Horticultural Sciences, Texas A&M University, College Station, TX, United States; ^2^Texas A&M AgriLife Research and Extension Center, Uvalde, TX, United States

**Keywords:** citrulline, drought stress, nitrogen, watermelon, arginine

## Abstract

A non-protein amino acid, citrulline, is a compatible solute involved in the maintenance of cellular osmolarity during abiotic stresses. Despite its significance, a coherent model indicating the role of citrulline during stress conditions has not yet emerged. We have used watermelon, naturally rich in citrulline, as a model to understand its accumulation during drought stress and nitrogen perturbation using transcriptomic and metabolomic analysis. Experiments were performed in the semi-controlled environment, and open field to study the accumulation of drought-induced citrulline in the vegetative tissues of watermelon by monitoring the stress treatments using physiological measurements. The amino acid profiling of leaves and stems in response to drought stress showed up to a 38 and 16-fold increase in citrulline content, respectively. Correlation between amino acids indicated a concomitant activation of a metabolic pathway that included citrulline, its precursor (ornithine), and catabolic product (arginine). Consistent with its accumulation, the gene expression analysis and RNA-Sequencing confirmed activation of citrulline biosynthesis-related genes – *Ornithine carbamoyl-transferase* (*OTC*), *N-acetylornithine deacetylase* (*AOD*) and *Carbamoyl phosphate synthases* (*CPS*), and down-regulation of catabolic genes; *Arginosuccinate lyase* (*ASL*) and *Arginosuccinate synthases* (*ASS*) in drought-stressed leaf tissues. Based on the relative abundance in the nitrogen-depleted vegetative tissues and down-regulation of genes involved in citrulline biosynthesis, we also demonstrated that the nitrogen status of the plant regulates citrulline. Taken together, these data provide further insights into the metabolic and molecular mechanisms underlying the amino acid metabolism under environmental stress and the significance of non-protein amino acid citrulline in plants.

## Introduction

Plants accumulate a variety of low molecular weight compatible solutes (osmolytes) in response to abiotic stresses. The most common osmolytes are glycine betaines, organic sugars, polyamines, and amino acids (proline, GABA). These osmolytes provide a range of protective functions such as maintenance of cellular osmotic adjustments and membrane integrity, stabilization of intrinsic proteins, buffering cellular pH, and detoxification of reactive oxygen species (ROS) (Hare et al., 1998; Chen and Murata, 2002; Slama et al., 2015; Ali et al., 2017). Although much research has been done to show the usefulness of common osmolytes (Lunn et al., 2014; Per et al., 2017; Romero et al., 2018; Annunziata et al., 2019), not all plants are capable of inducing the same set of osmolytes. The onset of these osmolytes varies considerably among plant species, tissue types, longevity, and nature of abiotic stresses.

Citrulline is an intermediate non-protein amino acid in the arginine pathway. In plants, it is synthesized from ornithine and carbamoyl phosphate. Citrulline is a potent scavenger of hydroxyl radicals and protects cellular enzymes from oxidative damage (Akashi et al., 2001, 2004; Yokota et al., 2002). The fruits of watermelon and other members of the *Cucurbitaceae* family accumulates large amounts of free citrulline (Davis et al., 2011; Fish, 2014; Akashi et al., 2017; Joshi et al., 2019). The accumulation of several amino acids during drought stress in different plant species has been reported (Obata and Fernie, 2012; Huang and Jander, 2017). However, limited studies have demonstrated accumulation of citrulline in the vegetative tissues in response to environmental stresses; such as watermelons (Kawasaki et al., 2000; Yokota et al., 2002; Akashi et al., 2008), melons (Mitchell and Madore, 1992; Mitchell et al., 1992; Daşgan et al., 2009; Kusvuran et al., 2013), chickpea (Garg et al., 2016; Khan et al., 2019). Even though the mechanism through which the drought stress leads to citrulline accumulation in plants is not completely understood. Accumulation of amino acids during drought stress has been generally attributed to decreased protein synthesis (Good and Zaplachinski, 1994), increased protein degradation (Huang and Jander, 2017; Hildebrandt, 2018), or changes in a gene network affecting biosynthesis or catabolism of substrate/product availability (Joshi et al., 2010; Hildebrandt et al., 2015). The metanalysis of metabolic and transcriptomic networks has demonstrated that the synthesis of abundant amino acids, such as proline, arginine, asparagine; is upregulated during abiotic stress (Hildebrandt, 2018; Sircar and Parekh, 2019) and these amino acids act as compatible osmolytes, precursors for secondary metabolites, or storage forms of organic nitrogen. In addition to amino acids, ureides such as allantoin, allanotoate, and citrulline are also used as nitrogen transporting molecules. Citrulline has also been suggested to play a role in facilitating nitrogen assimilation, endogenous nitrogen storage, and transport in higher plants (Reuter, 1961; Ludwig, 1993). The central role of citrulline as a molecular modulator for carbon and nitrogen integration into the urea cycle has been demonstrated in photosynthetic marine diatoms (Allen et al., 2011). However, little is known about its role in nitrogen metabolism in agriculturally important monocot or dicot crops. Nonetheless, citrulline has been long back proposed to function as a carrier of organic nitrogen in cucurbits (Kasting and Delwiche, 1958) and occurs at high concentrations in the phloem of cucurbits (Mitchell and Madore, 1992).

Being a naturally rich in citrulline and with the availability of resources such as published genome (Guo et al., 2013, 2015), next-generation sequence (NGS) data (Guo et al., 2015; Nimmakayala et al., 2016), and the databases^1^ [International Cucurbit Genomics Initiative (ICuGI)] watermelon would be an ideal model to study the drought-induced accumulation of citrulline in the vegetative tissues. Not much is known about the transcriptional or feedback regulation of citrulline in plants during environmental stresses in plants. The purpose of the proposed experiments is to provide new insights into the induction of citrulline and associated amino acids in vegetative tissues of watermelon and transcriptional regulation of citrulline during drought stress and nitrogen deficit. The outcome of the experiment validated the role of genes associated with the citrulline pathway and the significance of citrulline in drought stress and nitrogen deficit in plants.

## Materials and Methods

### Germination and Seedling Growth Condition

Commercial watermelon cultivars Crimson Sweet and Charleston Gray seeds were sown in 96-well plug trays (25′′ length × 15′ width × 2′ depth) in a greenhouse at the Texas A&M AgriLife Research & Extension Center at Uvalde, TX, United States. Additional details regarding the parentage, agronomic performance, and qualities of the two cultivars are available through the Cucurbit Breeding website at NC State^2^. The environmental factors inside the greenhouse were measured and monitored by a centralized control system (Wadsworth, Arvada, CO, United States) with 16 h light followed by 8 h dark. The temperature and humidity were maintained at 30 ± 5°C and 70 ± 5%, respectively.

### Drought Stress Experiment

For the drought stress experiment, ∼ 8-weeks old seedlings of the cultivar Crimson Sweet were transplanted to 10 heavy-duty round nursery pots (5 gallons; 28 cm width × 26 cm height) in a semi-controlled high-tunnel greenhouse at the Texas A&M AgriLife Research & Extension Center, Uvalde, TX, United States (Supplementary Figure S1). Before transplanting, all pots were filled with the same weight (∼22lb) of soil-less media Absorb-N-Dry (Balcones Minerals Corporation, Flatonia, TX, United States), saturated completely with water and left overnight for drainage. The weights of each pot were recorded to establish initial water content. All pots were applied with standard fertilizer mix to avoid nutritional imbalance (Peters Professional Mix 20:20:20). Decagon 5TE moisture sensors (Decagon Devices, Inc., Pullman, WA, United States) were inserted at a depth of 6 inches and connected to the EM 50 data loggers for recording, monitoring, and downloading the information of the percent volumetric water content (VWC) per pot. The 5TE sensor uses an electromagnetic field to measure the dielectric permittivity of the surrounding medium. The sensor supplies a 70 MHz oscillating wave to the sensor prongs that charges according to the dielectric of the material. The stored charge is proportional to soil VWC. The control (well-watered) pots were frequently watered to maintain 95 ± 5% of relative water content. In contrast, for drought stress treatment, the pots were subjected to stress by withholding watering 24 days after transplanting to keep the soil moisture at 35 ± 5% as per VWC data using daily readings recorded from the data loggers. The technique of freezing point depression was used for the determination of cell osmotic potential (ψs) using osmometer (Wescor Vapro osmometers 5520, Logan, UT, United States). Leaf disks (0.25-inch diameter) collected at 8, 24, and 53 days after induction of drought stress [days after initiation of treatment (DAT)] were wrapped in aluminum foils and flash-frozen in liquid nitrogen to measure ψs. When measuring the osmotic potential, leaf disks from control and stress plants were placed into the enclosed measuring chamber. Osmometer readings (mmol⋅kg^–1^) were converted to MPa as per the manufacturer’s instructions. The differences in the water potentials between the samples were analyzed using Tukey–Kramer HSD (*p*-value < 0.05). A gas analyzer (LI-6400 XT, LI-COR Bioscience, Lincoln, NE, United States) was used to measure leaf photosynthesis rate (Photo), stomatal conductance (Cond) and transpiration (Trmmol) with parameters (Photosynthetic Active Radiation, PAR,1000 μmol m^–2^ s^–1^; CO_2_ concentration, 400 ppm; leaf temperature, 20°C; VPD, 1 kPa; flow, 500 μmols^–1^) at 8 and 24 DAT to monitor the progressive changes in the photosynthetic performance due to drought stress. Tissue samples were collected from three to four independent plants for both amino acid analysis and RNA extraction at 8 DAT (Leaf-I and stem) and 24 DAT (Leaf II) and flash-frozen in liquid nitrogen for further analysis. Both Leaf-I and Leaf-II tissues were collected from the middle position of the main vines, while the stem sections were collected from the vine at a point where Leaf-I originated.

### Nitrogen (N) Deficit Experiment

Before initiating the N-deficit experiment, soil samples collected at 10 random locations at a depth of 6 inches were analyzed for elemental composition at Texas A&M AgriLife Extension Service Soil, Water and Forage Testing Laboratory^3^. Total Kjeldahl N (TKN) concentrations were estimated by the Kjeldahl method (Easy Chem Plus; Chinchilla Scientific, Oak Brook, IL, United States) with the addition of sulfuric acid for digestion in the presence of Kjeldahl formulated catalyst (Pro-Pac-CT 37; Alfie Packers, Inc., Omaha, NE, United States). Total inorganic nitrogen was calculated by adding the concentrations of soil nitrate, nitrite, and ammonia. Approximately 6-week-old seedlings of the two cultivars – Charleston Gray and Crimson Sweet were transplanted on the raised beds keeping plant to plant distance of 3 ft and rows to row spacing of 12 ft (Supplementray Figure S2). The beds were covered with plastic mulch and watered using sub-surface drip irrigation lines when needed depending on the air temperature and soil humidity. Based on the preliminary experiments (data not shown) and indigenous nitrogen levels, two N treatments, high N (HN) and low N (LN), were established to keep the N levels to 168 ± 11 kg/ha N and 44 ± 5 kg/ha, respectively. The nitrogen was applied in the form of Urea (46-0-0, HELENA, TX, United States). Soil samples were collected at a depth of 6-inches every week from the date of transplanting for soil elemental analysis to monitor the soil N content. The nitrate content of the plants was measured 10 weeks after transplanting using the petiole saps with portable Nitrate Pocket Tester (Horiba LAQUA twin NO_3_-11 Compact Water Quality Nitrate Ion Meter). The photosynthetic performance was measured 45, 59, and 69 DAT from the leaves located in the middle of the vines (data not shown). SPAD measurements were taken at 39, 45, 52, and 66 DAT. Tissue samples from both the varieties for amino acid analysis and RNA extraction were collected from three independent plants at 45, 59, and 69 days after the initiation of nitrogen stress treatment.

### Amino Acid Extraction and Quantification With UPLC-ESI-MS/MS

Approximately 20 mg fresh tissue samples were homogenized into a fine powder in a Harbil model 5G-HD paint shaker (Harbil, Wheeling, IL, United States) using 3 mm Demag stainless steel balls (Abbott Ball Company, West Hartford, CT, United States). Total free amino acids were extracted by suspending the homogenized samples in 10 μL of 100 mM cold HCl per mg of tissues, incubating on ice for around 20 min, and then centrifuging at a speed of 14,609 × *g* for 20 min at 4°C. The extracts were filtered through a 96-well 0.45-μm-pore filter plate (Pall Life Sciences, United States). The filtrates were used for derivatization using with AccQTag3X Ultra-Fluor kit (Waters Corporation, Milford, MA, United States) as per the manufacturer’s protocol. L-Norvaline (TCI AMERICA, United States) was used as an internal control. Amino acid calibrators were obtained from Kairos^TM^ Amino Acid Kit (Waters Corporation, Milford, MA, United States). Lyophilized powder of the mixture of amino acids calibrators was reconstituted in 0.1M HCl before derivatization. Calibration curves were built using TargetLynx^TM^ Application Manager (Waters Corporation, Milford, MA, United States). UPLC-ESI-MS/MS analysis was performed using Water’s Acquity H-class UPLC system equipped with Waters Xevo TQ mass spectrometer and electrospray ionization (ESI) probe. The Waters Acquity H-class UPLC system was composed of an autosampler, Waters ACQUITY UPLC Fluorescence (FLR) detector, and a CORTECS^TM^ UPLC C_18_ (1.6 μm, 2.1 mm × 150 mm). The mobile phase was composed of (A) water (0.1% formic acid v/v) and (B) acetonitrile (0.1% formic acid v/v). The column heater was set at 60°C, and the mobile phase flow rate was maintained at 0.5 mL/min. The gradient of non-linear separation was set as follows: 0–1 min (99% A), 3.2 min (87.0% A), 8 min (86.5% A), and 9 min (5% A). Two microliters of the derivatized sample were injected for analysis. Multiple Reaction Monitoring (MRM) transitions and collision energy values and cone voltage were optimized for each amino acid using the Water’s IntelliStart software. The ESI source was operated at 150°C, gas desolvation flow rate at 1000 L/h, gas flow cone at 20 L/h, desolvation temperature at 500°C, for detecting all amino acids. MRM was performed in a positive mode. Water’s MassLynx software was used for instrument monitoring and data acquisition. The data integration and quantitation were carried out using Waters TargetLynx software.

### RNA-Seq Analysis of Drought Stress Leaves

#### RNA Extraction and Library Preparation for Transcriptome Sequencing

The flash-frozen leaf samples (Leaf-I) collected 8 DAT from three independent plants along with control (well-irrigated) plants were used for RNA-Seq analysis. Total RNA was extracted using a Quick-RNA Miniprep Kit (Zymo Research Corporation, Irvine, CA, United States) as per the manufacturer’s protocol and treated with DNase1 (Zymo Research Corporation, Irvine, CA, United States). The purity of the RNA was analyzed using the NanoPhotometer spectrophotometer (IMPLEN, Westlake Village, CA, United States). RNA integrity and quantitation were assessed using the RNA Nano 6000 Assay Kit of the Bioanalyzer 2100 system (Agilent Technologies, Santa Clara, CA, United States). One μg total RNA per sample was used for the RNA library preparations. Sequencing libraries were generated using NEBNext Ultra RNA Library Prep Kit for Illumina (NEB, United States) following the manufacturer’s recommendations, and index codes were added to attribute sequences to each sample. Library concentration was first quantified using a Qubit 2.0 fluorometer (Life Technologies), diluted to 1 ng/μl before checking the insert size on an Agilent Bioanalyzer 2100 system and quantified to greater accuracy by quantitative PCR (Q-PCR) (library activity >2 nM).

#### Data Processing, Analysis, and Mapping to Reference Genome

The clustering of the index-coded samples was performed on a cBot Cluster Generation System using PE Cluster Kit cBot-HS (Illumina) according to the manufacturer’s instructions. After cluster generation, the libraries were sequenced on an Illumina Hiseq platform, and 150 bp paired-end reads were generated. Raw reads of fastq format were processed to obtain clean reads by removing the adapter, reads containing ploy N, and low-quality reads from raw data. At the same time, Q20, Q30, and GC content, the clean data were calculated. Watermelon reference genome (cultivar Charleston Gray) and gene model annotation files were downloaded from CuGenDB^1^. Index of the reference genome was built using Bowtie v2.2.3, and paired-end clean reads were aligned to the reference genome using TopHat v2.0.12.

#### Gene Expression Quantification and DEG Analysis

HTSeq v0.6.1 was used to count the reads mapped to each gene. FPKM (Trapnell et al., 2010) of each gene was calculated based on the length of the gene and reads count mapped to this gene. Differential expression analysis of control and drought-stressed conditions (three biological replicates per tissue per treatment) was performed using the DESeq R package (Anders and Huber, 2010). Genes with *p*-value < 0.05 found by DESeq were assigned as differentially expressed.

### Real-Time Polymerase Chain Reaction

Total RNA was extracted with the Quick-RNA Miniprep Kit (Zymo Research Corporation, Irvine, CA, United States) treated with DNase1 (Zymo Research Corporation, Irvine, CA, United States), and subjected to reverse transcription using iScript RT Supermix (Bio-Rad Laboratories, Inc., Hercules, CA, United States). The quality and quantity of the RNA were analyzed by a Denovix DS-11+ spectrophotometer (Wilmington, DE, United States). Gene expression analysis via reverse transcription-qPCR was performed in the BioRad CFX96 qPCR instrument using SsoAdv Univer SYBR GRN Master Kit (Bio-Rad Laboratories, Inc., Hercules, CA, United States). Relative gene expression levels were determined using a standard curve method, and the value for each target gene was then normalized against the mean of expression values of the watermelon β-actin and α-tubulin5 genes (Kong et al., 2014) as reference genes. The stability of both the reference genes was confirmed using BestKeeper (Pfaffl et al., 2004). The relative expression levels (Cq values) for each gene were normalized to that of reference genes by taking an average of three biological replicates. The relative expression levels were calculated using the ΔΔCq (quantitative cycle) method provided with the Bio-Rad CFX software. Primers for qPCR used in the study and the BestKeeper analysis of expression stability of reference genes are listed in Supplementary Table S1.

## Results

### Validation of Drought Stress Treatment

**FIGURE 1:**
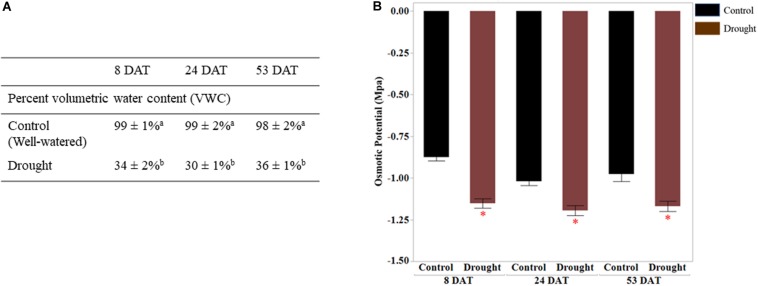
Validation of drought stress treatment. **(A)** Percent volumetric water content (WVC) was measured 8, 24, and 53 days DAT using 5TE sensors. The percent VWC data represent the ratio of actual water volume to maximum water volume (water content in saturated soil pot). The values are means ± SD (*n* = 3 or 4) and the different letters represents significance between control and drought treatments at *p* < 0.05. **(B)** Osmotic potential (Mpa) of leaf tissues measured at three different time points is shown on vertical axis. The values represent means ± SD (*n* = 3 or 4) and asterisk (^∗^) represents significant difference between control and drought treatments (*p* < 0.05). DAT, days after initiation of drought treatment.

**FIGURE 2:**
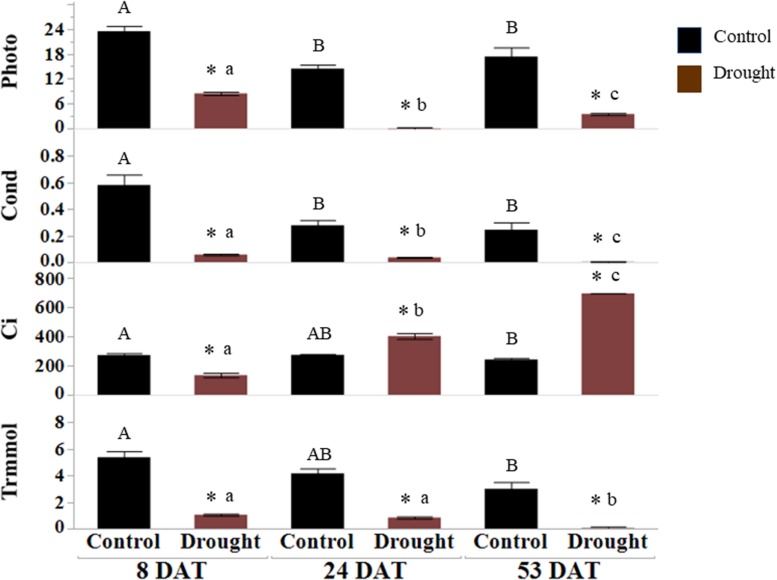
Gas analyzer measurements of drought-stressed leaves. Net photosynthesis (Photo, mol CO_2_ m^–2^ s^–1^), stomatal conductance (Cond, mol H_2_O m^–2^ s^–1^), intercellular CO_2_ (C_i_, μmol CO_2_ mol air^–1^) and transpiration rate (Trmmol, mol m^–2^ s^–1^) were measured at 8, 24, and 53 DAT. The values represent means ± SD (*n* = 3 or 4), asterisk (^∗^) represents significant difference between control and drought treatments, upper case letters between controls and lower-case letters between the drought treatments across DATs (*p* < 0.05). DAT, days after initiation of drought treatment.

Soil moisture and leaf osmotic potential: before transplanting seedlings into pots, 5TE probes inserted at a depth of root zones were calibrated by correlating readings (m^3^/m^3^) from the data loggers with actual water content (%) in the pots. The percent VWC measured by sensors at 8, 24, and 53 DAT is presented in Figure 1A. All pots receiving drought stress treatment were maintained at 35 ± 5% water content, implying consistent drought stress. The water content for the control (well-watered) pots was above 95 ± 5%. Osmotic potential (ψs) was measured with a VAPRO 5520 vapor pressure osmometer (Wescor, Logan, UT, United States) (Ball and Oosterhuis, 2005). Ψs is a negative quantity of osmotic pressure, and it decreases during dehydration due to the concentrated solutes in plant cells. The significantly consistent differences in the osmotic pressure (MPa) of leaf samples collected from control (well-watered) and drought-stressed plants were seen at all time points since the initiation of drought stress (Figure 1B). The net rates of photosynthesis (Photo), stomatal conductance (Cond), and transpiration rates (Trmmol) in drought-stressed plants were significantly lower than the control plants (Figure 2). Decrease of the intercellular CO_2_ concentration (Ci) at the beginning of drought stress (8 DAT) indicates that photosynthesis is limited more by the decrease of stomatal conductance.

### Drought Stress-Induced Accumulation of Citrulline and Other Amino Acids

**FIGURE 3:**
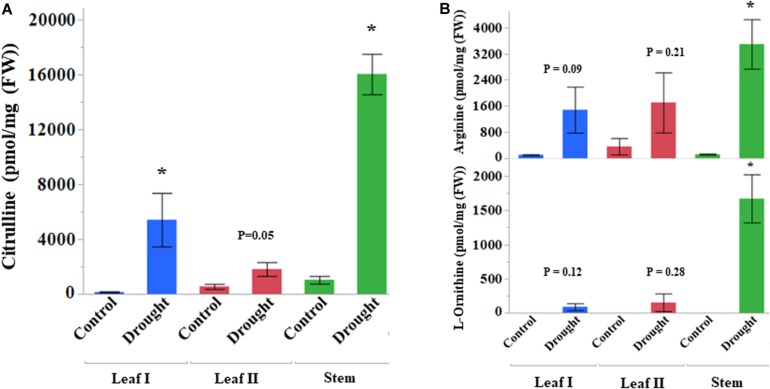
Drought stress induced accumulation of amino acids (pmol/mg FW). Vegetative tissues showing changes in the content of **(A)** Citrulline and **(B)** Ornithine and Arginine. DAT, date after treatment. Leaf I and Stem tissues collected at 8 DAT, Leaf II at 24 DAT, Asterisk (^∗^) indicates significance from control at respective DAT between control and drought treatments at *p* < 0.05 based on Student’s *t*-test. Values are mean ± SD (*n* = 3 or 4) from four independent replications. DAT, days after initiation of drought treatment.

Citrulline contents in all the stressed samples after the initiation of the drought stress increased dramatically (Figure 3A). At the onset of drought stress treatment, citrulline content in the leaf and stem enhanced rapidly – up to 38 and 16-fold, respectively (Supplementary Table S2). The leaf tissue collected during sustained drought stress (24 DAT) also showed threefold increases in citrulline. Although non-significant, the content of both ornithine (precursor of citrulline, *p* = 0.09) and arginine (catabolic product of citrulline, *p* = 0.12) (Figure 3B) also showed increasing trends at least during early drought stress (8 DAT, Leaf I). Nonetheless, their increases in stem tissue were consistent with the fold changes in citrulline. The correlation analysis of amino acids detected from leaf-I at 8 DAT in drought-stressed plants showed a significant positive correlation between ornithine, citrulline, and arginine (Supplementary Figure S3). The content of other amino acids, such as phenylalanine, valine, proline, glycine, serine, glutamine, and glutamic acid was at least twofold higher in drought-stressed leaf tissue at 8 DAT. Most amino acids in stem showed many-fold increases during drought stress than control tissues (Supplementary Table S2). In terms of percent distribution, citrulline alone accounted for almost 25 and 21% of total amino acids at 8DAT in the drought-stressed leaf and stem tissues respectively (Supplementary Figures S4, S5).

### Expression of Genes Involved in Citrulline Metabolism Using RNA-Seq Analysis of Leaf

#### Transcriptome Sequencing and Assembly of Sequencing Data

To understand the early transcriptomic changes induced due to drought stress, we performed RNA-Sequencing (RNA-Seq) analysis of leaf tissue 8 days after the initiation of drought stress. A total of six libraries from leaf tissue (CT_1, CT_2, CT_3 for control and DT_1, DT_2, DT_3 for drought stress) were sequenced using the Illumina HiSeq platform. On average, 45.53 to 43.47 million raw reads were generated from leaf tissues in both treatments (Supplementary Table S3). Across all reads for both treatments, the Q20 and Q30 percentage was more than 98 and 94%, respectively (sequencing error rate was less than 0.02%), and GC content for the libraries was ∼45%. Additional significant characteristics of these libraries are summarized in Supplementary Table S3. Among all the libraries, the ratio of total mapped reads was above 97%, of which ∼92% reads uniquely mapped to the reference watermelon genome. The data generated from all libraries provided a foundation for quality analyses. The correlations among biological replicates were assessed using the Pearson correlation coefficient (Supplementary Figure S6). The libraries for the same treatment were highly correlated. The weak correlation between treatments suggests a significant impact of drought stress on gene expression profiles.

### Analysis of Differentially Expressed Genes (DEGs) Associated With Citrulline Metabolism

**FIGURE 4:**
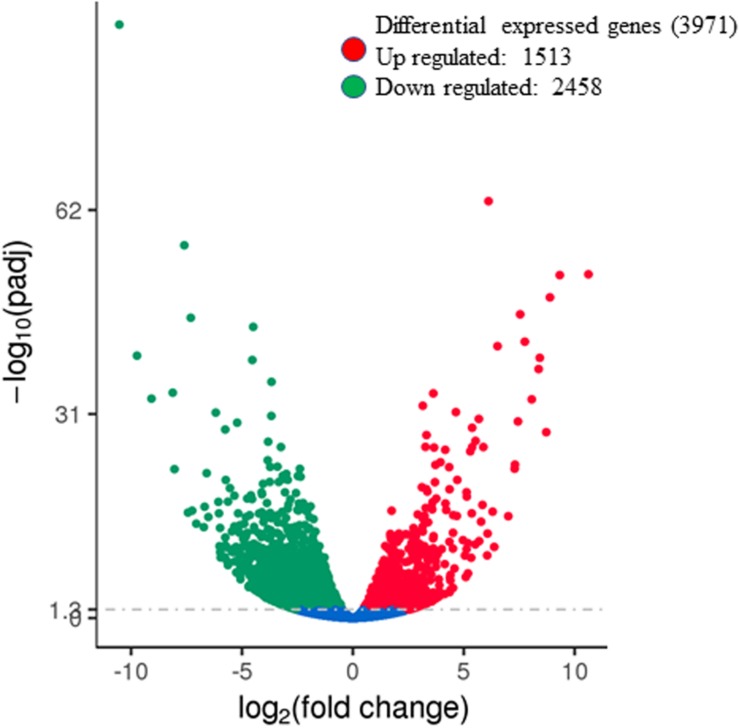
Summary of differentially expressed genes during drought stress. The volcano map showing the number of differentially expressed genes in the leaf tissue (8 DAT) due to drought stress. Red dots represent up-regulated genes and green dots represent down-regulated genes (padj < 0.05). Blue dots indicate no significant difference in genes padj(qvalue) is corrected *p*-value. DAT, days after initiation of drought treatment.

The RNA-Seq analysis identified 3971 differentially expressed (*p* < 0.05) genes in the leaf tissue of drought-stressed plants. The volcano map showing a total of 1513 upregulated genes and 2458 downregulated genes is presented in Figure 4. The list of genes showing up- and down-regulation of genes due to drought stress are listed in Supplementary Tables S4, S5, respectively. The fold-change differences in the expression of a subset of genes associated directly or indirectly with citrulline metabolism are presented in Table 1. The transcript quantification confirmed significant increases in the expression of *N-acetylornithine deacetylases* (*AOD*, ClCG09G012030, ClCG09G012020), the large subunit of Carbamoyl *phosphate synthase* (*CPS2*, ClCG09G021680), and *N-acetylornithine aminotransferase* (ClCG09G003180) which all are associated directly with citrulline synthesis. Expression of all three *Arginosuccinate synthases* (*ASS*; ClCG03G003660, ClCG03G003670, and ClCG06G017780) involved in two-step degradation of citrulline was significantly downregulated. Similarly, the expression of *Ornithine decarboxylase* (*ODC*; ClCG08G013990) associated with the alternative pathway of ornithine catabolism was also significantly downregulated.

**TABLE 1:** RNA-Seq data showing fold-change differences in the expression of genes associated with citrulline metabolism.

Gene IDs	Gene function	log2 fold-change	*p*-Value
C1CG09G012030	*N*-acetylornithine deacetylase	7.44	0.00
C1CG09G021680	Carbamoyl phosphate synthase, large subunit	1.84	0.00
C1CG09G012020	*N*-acetylornithine deacetylase	1.14	0.00
C1CG04G004210	Pyrroline-5-carboxylate reductase	0.82	0.04
C1CG01G004960	Nitric-oxide synthase, putative	0.81	0.01
C1CG09G003180	*N*-acetylornithine aminotransferase	0.79	0.01
C1CG03G006680	Ornithine-δ-aminotransferase	0.71	0.00
C1CG05G018820	Ornithine carbamoyltransferase	0.67	0.07
C1CG08G011660	Argininosuccinate lyase	0.65	0.09
C1CG11G003830	Arginine decarboxylase	0.63	0.01
C1CG01G014440	P5C dehydrogenase	0.5	0.03
C1CG06G000480	Arginase	0.38	0.32
C1CG01G017650	*N*-acetyl Glu synthase	0.09	0.82
C1CG11G013120	Carbamoyl phosphate synthase, small subunit	–0.22	0.42
C1CG08G012830	*N*-acetylglutamate kinase	–0.24	0.50
C1CG09G004810	P-II	–0.24	0.33
C1CG09G012040	*N*-acetylornithine deacetylase	–0.35	0.72
C1CG10G020940	*N*-acetylornithine-glutamate acetyltransferase	–0.36	0.36
C1CG11G014580	Argininosuccinate lyase	–0.42	0.08
C1CG11G013850	Urease	–0.59	0.12
C1CG11G005180	Proline dehydrogenase	–0.92	0.14
C1CG09G017590	*N*-acetyl-γ-glutamyl-P-reductase	–1.02	0.06
C1CG10G021450	Pyrroline-5-carboxylate synthase	–1.08	0.09
C1CG03G003660	Argininosuccinate synthase	–1.16	0.02
C1CG06G017780	Argininosuccinate synthase	–1.58	0.00
C1CG11G006600	Pyrroline-5-carboxylate synthase	–1.67	0.00
C1CG03G003670	Argininosuccinate synthase	–2.25	0.00
C1CG08G013990	Ornithine decarboxylase	–2.49	0.01

**FIGURE 5:**
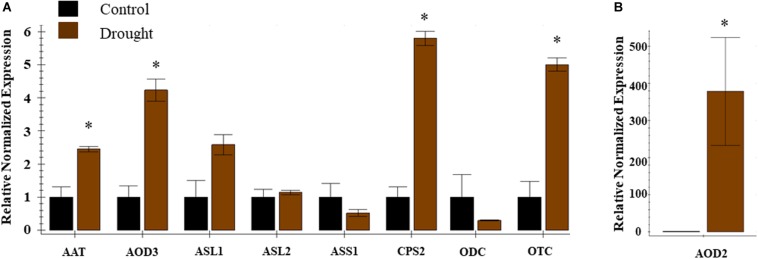
Expression profiles of genes associated with citrulline metabolism during drought stress. Real time expression analysis was carried out in leaf tissue 8 DAT from control and drought stressed plants. The error bars represent the means ± SE (*n* = 3), asterisk indicates significant differences in the expression of a specific gene compared to control sample (*p* < 0.1). Relative normalized expression is presented for nine **(A)** genes viz. AAT, *N-*acetylomithine transferase; AOD3, *N-acetylornithine* deacetylase; ASL-1, 2 Arginosuccinate lyase; ASS-1, Arginosuccinate synthase; CPS2, Carbamoyl phosphate synthase -large subunit; OCD, ornithine cyclodeaminase; ODC, Omithine decarboxylase; OTC, Omithine transcarboxylase; DAT, days after initiation of drought treatment and **(B)** AOD2, *N*-acetylornithine deacetylase.

Ten DEGs associated with citrulline metabolism were used for real-time qRT-PCR analysis to validate the RNA-Seq data. The expression analysis confirmed the induction of *CPS2*, *Ornithine carbamoyltransferase (OTC*), *AOD2*, *AOD3*, and *N-Acetylornithine aminotransferase* (*AAT*) and trend for down-regulation of *ASS1* (Figure 5). The qPCR expression data confirmed the results and reproducibility of the RNA-Seq data for the subset of selected genes (Supplementary Figure S7).

### Impact of Nitrogen Deficiency on Citrulline Metabolism

#### Validation of Nitrogen Deficit Treatment

A field experiment was performed to evaluate the impact of nitrogen deficit on citrulline accumulation in the vegetative tissues during development. The nitrogen deficit was validated based on significantly lower total soil inorganic nitrogen (Supplementary Figure S8A) and free nitrates (Supplementary Figure S8B). Additionally, the estimates of nitrate in the phloem sap (Supplementary Figure S9), total nitrogen in leaves (Supplementary Figure S10), and chlorophyll content (Supplementary Figure S11) further confirmed the compromised nitrogen assimilation in the plants under nitrogen deficit.

#### Changes in the Citrulline Content and Gene Expression During Nitrogen Deficit

**FIGURE 6:**
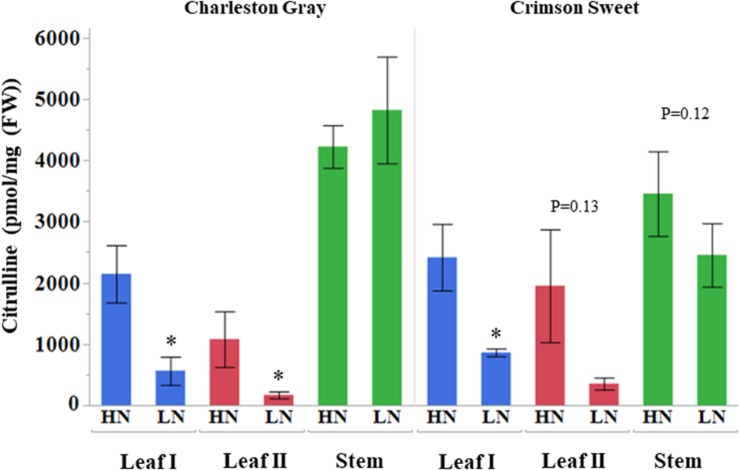
Citrulline accumulation in watermelon tissues during nitrogen deficit (pmol/mg FW). Citrulline content of plant tissues collected during early 45 DAT (Leaf I and stem) and extended 69 DAT (Leaf II) nitrogen deficit treatment is shown on vertical axis. The values represent means ± SD (*n* = 3 or 4). The asterisk (^∗^) represent significant differences between high (HN) and nitrogen deficit (LN) treatments (*p* < 0.1, Student’s *t*-test). DAT, days after initiation of nitrogen deficit treatment.

**FIGURE 7:**
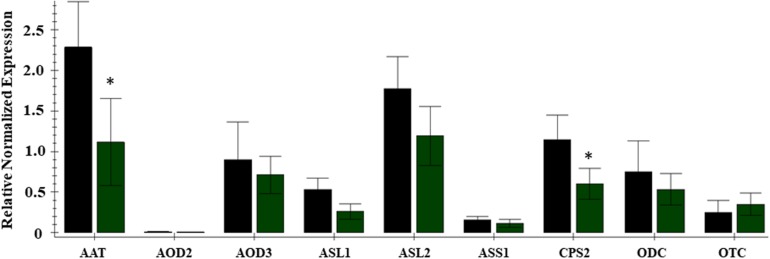
Expression analysis of genes associated with citrulline metabolism during nitrogen deficit in the cultivar Crimson Sweet. Real-time expression analysis was carried out using leaf tissues 45 DAT from plants subjected to nitrogen stress LN (green bars) and control HN (black bars). The error bars represent the means ± SE (*n* = 3); an asterisk indicates significant differences in the expression of a specific gene compared to the control sample (*p* < 0.1) for nine genes viz. *AAT*, *N-acetyrlornithine transferase*; *AOD3*, *N-acetylornithine deacetylase*; *ASL-1*, *2Arginosuccinate lyase*; *ASS-1*, *Arginosuccinate synthase*; *CPS2, Carbamoyl phosphate synthase -large subunit*; *ODC, Omithine decarboxylase*; *OTC, Ornithine transcarboxylase*; *AOD2, N-acetylomithine deacetylase*; DAT, days afterinitiation of drought treatment.

Citrulline content in the leaf tissues was altered significantly, vis-à-vis the availability of nitrogen. The citrulline in leaf tissues collected 45 and 69 days after initiation of nitrogen stress was reduced by 5 to 6-fold in cultivar Charleston Gray and 2 to 6-fold in Crimson Sweet (Figure 6). Ornithine content was only detectable in leaves during the early stages of plant development and was reduced by several folds in both the cultivars due to nitrogen stress (Supplementary Tables S6, S7). While arginine content was down by eightfold due to nitrogen stress in the cultivar Crimson Sweet at 45 days after the initiation of nitrogen stress. Besides, aspartate and glutamine, which reduced significantly due to nitrogen stress, most other amino acids showed a trend for reduced accumulation due to nitrogen stress in leaf tissues. The changes in amino acids were not consistent in the stem of both the cultivars. In terms of relative contribution, percent distribution of citrulline, arginine, or ornithine relative to all amino acids was significantly reduced due to nitrogen stress in the vegetative tissues of both the cultivars (Supplementary Figures S12, S13). Further, we also evaluated changes in the content of amino acids in the phloem sap due to nitrogen stress. Although differences were insignificant in the case of cultivar Charleston Gray, the amounts of citrulline, arginine, and ornithine were significantly reduced in the cultivar Crimson Sweet subjected to nitrogen stress (Supplementary Figure S14). A subset of genes associated with citrulline metabolism was evaluated using the quantitative real-time gene expression analysis of leaf tissue using cv. Crimson Sweet (Figure 7). The expression of *AAT* and *CPS2* associated with citrulline biosynthesis were downregulated. Unlike the drought-stressed plants, the expression of other genes associated with citrulline metabolism was mostly unchanged due to nitrogen stress.

## Discussion

### Induction of Citrulline in the Vegetative Tissues of Watermelon During Drought Stress

Abiotic stresses such as drought stress or sub-optimal nitrogen levels, severely limit the productivity of many crops. Tolerance to abiotic stresses is a complex trait requiring orchestrated regulation of physiological, molecular, and biochemical responses, each making incremental contributions. Exposure to drought stress disrupts the electron transport chains resulting in the accumulation of ROS, which can lead to cellular damage. Amino acids induced in response to abiotic stresses act as powerful ROS-scavengers or antioxidants (Verslues and Juenger, 2011). Being potentially potent hydroxyl radical scavenger than other compatible solutes like mannitol, proline, and glycine betaine (Akashi et al., 2001; Yokota et al., 2002), citrulline contributes to protecting green tissues from the secondary oxidative stress damage during drought stress. Although produced in large amounts in the fruits of cucurbits, not much is known about the physiological or biochemical relevance of citrulline in the vegetative tissues of plants. Citrulline has been proposed to play a broader role in abiotic stress tolerance as an active osmolyte, nitrogen carriers, and possibly a signaling molecule (Joshi and Fernie, 2017). Our results demonstrated a rapid early induction (∼38 fold) of citrulline within 8 days of initiation of drought stress. Citrulline accumulation in leaf tissues further increased during prolonged stress, but its induction relative to control decreased (∼16 fold). The early rapid induction of citrulline implicates its utility as a potential biomarker for drought-induced responses. The fold-change increases in arginine (catabolic product of citrulline) and ornithine (precursor), consistent with the increase in citrulline and their high positive correlations with one another, emphasize the significance of the ornithine-citrulline-arginine pathway in watermelons during drought stress. Nonetheless, the importance of the glutamate-derived arginine pathway in abiotic stress tolerance in higher plants is well-supported, due to the production of stress-induced intermediates such as ornithine, citrulline, nitrous oxide, polyamines and proline (Kalamaki et al., 2009; Winter et al., 2015). Further, in terms of abundance, percent citrulline accumulation due to drought stress varied from 21 to 25% in stem and leaf tissues, respectively. These findings are consistent with a report showing almost 49% accumulation of citrulline during drought stress in the leaves of *Citrullus colocynthis*, a wild relative of watermelon (Kawasaki et al., 2000; Yokota et al., 2002). Transgenic approaches such as overexpression of DOF transcription factor (Massange-Sánchez et al., 2016) and *N*-acetyl-L-glutamate synthase, a first biosynthetic enzyme in the arginine pathway (Kalamaki et al., 2009) in Arabidopsis have demonstrated a positive association between increased citrulline and drought tolerance. Enhanced citrulline accumulation and possibly genes associated with its metabolism in watermelon could provide cues to manipulate citrulline concentrations in plant species that do not accumulate enough citrulline.

### Transcriptional Regulation of Citrulline Metabolism

The complex nature of plant responses to drought stress is evident by thousands of differentially expressed genes identified in numerous transcriptomic datasets available in the public domain. However, a coherent picture of a generalized regulation of citrulline metabolism based on gene expression profiles across plant species have not yet emerged. In this study, using the genome sequence of cultivar Charleston Gray, we identified 3971 differentially expressed genes in drought-stressed leaves of watermelon, out of which 132 were novel genes. Our transcriptome data indicated that most genes related to citrulline metabolism were differentially expressed under drought stress (Table 1). Notably, the expression of *N-acetylornithine deacetylases*, *Carbamoyl phosphate synthase (large subunit), N-acetylornithine aminotransferase*, and *OTC* directly associated with citrulline synthesis were significantly up-regulated in response to drought stress, which is consistent with the enhanced levels of citrulline and arginine. Moreover, the expression of *Argininosuccinate lyase (ASL2)*, and all three *Argininosuccinate synthase* genes associated with citrulline catabolism; and expression of *ODC* involved in ornithine catabolism were significantly downregulated. However, the activation of citrulline biosynthetic genes is consistent with RNAseq profiles of the drought-stressed leaves of *C. colocynthis* plants (Wang et al., 2014).

**FIGURE 8:**
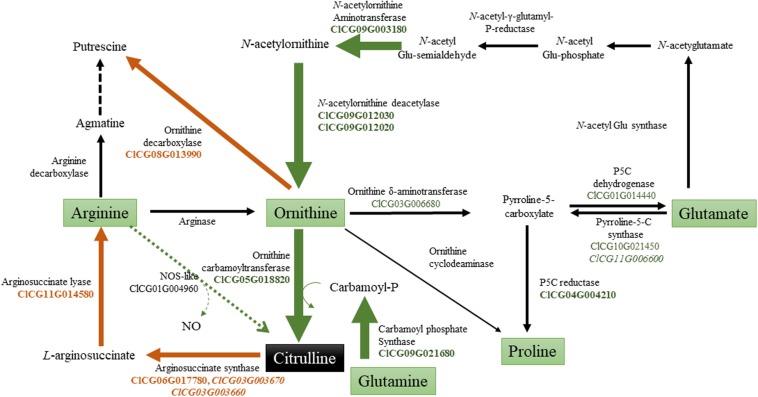
Model showing the regulation ofcitrulline metabolism during drought stress. Based on expression and RNA-Seq analysis, genes involved in citrulline metabolism were grouped in into biosynthetic and catabolic genes. The green arrows indicates induction of genes associated with citrulline synthesis; while brown arrow’s down-regulation of genes involved in citrulline/ornithine degradation [Gene EDs from Watermelon genome (cultivar Charleston Gray) [ntemational Cucurbit Genomics Initiative].

A model illustrating the possible regulation of citrulline during drought stress by activation of biosynthetic genes (*AAT, AOD, OTC, and CPS2*) and down-regulation of catabolic (*ASS, ASL, ODC*) genes is presented (Figure 8). Among the biosynthetic genes, expression of *AAT* which catalyzes the reversible conversion of *N*-acetylglutamate 5-semialdehyde to *N*-acetylornithine in the presence of glutamate, was highly induced in drought-stressed leaves. *N*-acetylornithine aminotransferase in Arabidopsis (Frémont et al., 2013) is highly expressed in leaves and involved in arginine synthesis. Consistent with the induced expression of *N-acetylornithine deacetylases* genes in the drought-stressed leaves of *C. colocynthis* (Wang et al., 2014), both the *AOD2* (ClCG09G012030) and *AOD3* (ClCG09G012020) genes were induced in the leaf tissue in response to drought stress. In Arabidopsis, AOD is involved in ornithine synthesis and regulation of the C: N balance during reproduction via polyamine synthesis (Molesini et al., 2015). It was previously suggested that wild watermelon accumulates high levels of acetylornithine deacetylase (*DRIP1; ArgE*) transcripts (Kawasaki et al., 2000) and *N*-acetylornithine: *N-*acetylglutamate acetyltransferase (*NAOGAcT*) (Takahara et al., 2005) during drought stress. Our RNA-Seq analysis did not show any change in the expression of *NAOGAcT* (ClCG10G020940), which is involved in the non-linear pathway of ornithine/citrulline synthesis (Joshi and Fernie, 2017). Instead, synthesis of citrulline through the linear pathway *via* induction of *AAT* and both *AOD* genes justifies the superfluous energy-intensive regeneration of *N*-acetylglutamate through the non-linear pathway during drought stress. OTC and carbamoyl phosphate synthase (CPS2) catalyzing the first and penultimate steps in citrulline synthesis were both induced due to drought stress aligning with the increased citrulline phenotype in leaf tissues. The indispensable role of these genes in citrulline synthesis is apparent based on reduced citrulline production in the leaves of ven*6* knock-down mutation in the large subunit of Arabidopsis CPS (Mollá-Morales et al., 2011).

The expression of *ASS1* (ClCG06G017780), *ASS2* (ClCG03G003670), *ASS3* (ClCG03G003660), and *ASL2* (ClCG11G014580) genes, involved in citrulline catabolism, were significantly down-regulated corroborating the increased citrulline accumulation in drought-stressed leaves. Although *ASS* and *ASL* genes are critical for the synthesis of arginine and downstream products such as polyamines, not much is known with respect to their functional significance during abiotic stress in plants. Even So, the developmental down-regulation of *ASS* and *ASL* genes has been shown to increase citrulline concentration in maturing watermelon fruits (Guo et al., 2013, 2015; Joshi et al., 2019). The existence of a pathway synthesizing citrulline from arginine via *nitric oxide synthase* (*NOS*) still awaits an experimental confirmation in plants (Moreau et al., 2010; Santolini et al., 2017). Although our data shows the induction of a *NOS*-like (ClCG01G004960) gene, its potential role in citrulline production remains uncertain without its functional validation. Even if ODC is well-studied for its role in polyamine synthesis and abiotic stresses tolerance (Chen et al., 2019), expression of *ODC* gene (ClCG08G013990) was down-regulated in drought-stressed leaf tissue. Given, the ODC and OTC enzymes compete for the common pool of ornithine, down-regulation of *ODC* substantiates the importance of increased citrulline accumulation in drought-stressed tissues in watermelon. Although the downregulation of the ClCG05G008460 gene annotated as *Ornithine cyclodeaminase*^1^ justifies the increased citrulline, the enzyme lacks functional validation and shares low homology (42%) with canonical Ornithine cyclodeaminases. Intriguingly, RNA-Seq data also identified the induction of *Ornithine* δ-*aminotransferase*, *Pyrroline-5-carboxylate synthase* (P5CS), and *Pyrroline-5-carboxylate reductase*, major genes involved in the synthesis of proline. Relative to citrulline, fold-change increase, or percent distribution of proline was minimal. It is plausible to assume that proline and citrulline act synergistically, although proline may play a subordinate role in drought tolerance in watermelon.

### Impact of Nitrogen Limitation on Citrulline Metabolism

Although less common than allantoin, it was suggested that nitrogen-rich citrulline could represent a significant portion of the endogenous source of nitrogen storage and transport (Ludwig, 1993). Citrulline has been a mode of transport of nitrogen in legumes (Parsons and Sunley, 2001) xylem saps of trees (Cramer et al., 2002; Frak et al., 2002) and in storage tissues in walnut (Mapelli et al., 2001). Our data demonstrated that citrulline synthesis is regulated by the nitrogen status of the plant. The results (1) significant reduction in citrulline content along with ornithine or arginine in the leaf tissues (2) the percent distribution in the nitrogen depleted leaves and stem (10–18%), comparable to that of nitrogen carrying amino acids – aspartate and glutamate (3) reduction in the phloem sap (cv. Crimson Sweet), during nitrogen stress and (4) down-regulation of biosynthetic genes *AAT* and *CPS2*, all support the role of citrulline in translocating nitrogen in the vegetative tissues of watermelon. Consistent with our results seedlings of a loss-of-function mutant of Arabidopsis nitrogen regulatory protein P-II, a gene located upstream in the citrulline metabolic pathway (Joshi and Fernie, 2017), grown on nitrogen depleted media accumulated up to 70% less citrulline along with down-regulation of *CPS* expression (Ferrario-Mery et al., 2006), while its over-expression in *Lotus japonicus* resulted in a 10-fold increase in free citrulline in nodules under nitrogen replete conditions (D’apuzzo et al., 2015). The data showing reduced levels of citrulline in the phloem sap further supports the role of citrulline in nitrogen translocation or long-distance transport. Consistent with this observation, citrulline was shown to be a significant component of phloem sap in muskmelon (*Cucumis melo* L) (Mitchell et al., 1992). The drought-induced *N*-acetyl glutamate transferase-like protein – DRIP-1 (Kawasaki et al., 2000) involved in citrulline synthesis was initially identified in the phloem exudates (Yokota et al., 2002). Moreover, the phloem sap transcriptome of watermelon (Guo et al., 2013) indicated a 35-fold higher expression of the *AOD* gene, which also supports the possibility of localized citrulline synthesis in the sieve elements for its long-distance transport. The differences in the amino acid composition of phloem sap among cultivars could be attributed to the genotypic or environmental factors.

## Conclusion

This study illustrated the significance of citrulline metabolism during abiotic stresses such as drought and nitrogen limitation in watermelon. The drought-induced rapid accumulation of citrulline and related metabolites in the vegetative tissues was demonstrated. Although the metabolic consequences of citrulline accumulation during drought stress are not fully understood, our findings show how the metabolic pathways associated with citrulline synthesis and catabolism are regulated in the vegetative tissues of watermelon during drought stress. The gene expression analysis indicated that citrulline perturbation due to drought stress is regulated simultaneously by activation of its biosynthesis and suppression of its catabolism. The RNA-Sequencing data presented in this study, which characterized the differentially expressed genes during drought stress, would serve as a resource to understand transcriptomic mechanisms induced by drought stress. Our data also demonstrated that the nitrogen status of the plant regulates citrulline synthesis. A detailed analysis of citrulline metabolism in model species using loss-of-function mutants and metabolic flux analysis would allow a better understanding of rate-limiting stages in citrulline synthesis, and its functional relevance in abiotic stresses. The outcome of this study will not only improve the current understanding of citrulline metabolism in agriculturally important crop – watermelon but vertically advance the knowledge about its metabolism in plants that do not accumulate citrulline to facilitate metabolite engineering.

## Data Availability Statement

The datasets generated for this study are deposited in NCBI’s Gene Expression Omnibus (Edgar et al., 2002) and are accessible through GEO Series accession number GSE144814, https://www.ncbi.nlm.nih.gov/geo/query/acc.cgi?acc=GSE144814.

## Author Contributions

QS and VJ conceived and designed the work and wrote the manuscript. QS, MJ, and JD performed the experiments. QS, VJ, and MJ analyzed the data. VJ, QS, and MJ revised the manuscript critically.

## Conflict of Interest

The authors declare that the research was conducted in the absence of any commercial or financial relationships that could be construed as a potential conflict of interest.

## Footnotes

1 http://cucurbitgenomics.org/

2 http://cucurbitbreeding.com/watermelon-breeding/watermelon-cultivars-ca-cz/

3 http://soiltesting.tamu.edu
